# A Cross-Cultural Comparison of Health Behaviors between Saudi and British Adolescents Living in Urban Areas: Gender by Country Analyses

**DOI:** 10.3390/ijerph10126701

**Published:** 2013-12-03

**Authors:** Hazzaa M. Al-Hazzaa, Yahya Al-Nakeeb, Michael J. Duncan, Hana I. Al-Sobayel, Nada A. Abahussain, Abdulrahman O. Musaiger, Mark Lyons, Peter Collins, Alan Nevill

**Affiliations:** 1 Pediatric Exercise Physiology Research Laboratory, College of Education, King Saud University, P.O. Box 2458, Riyadh 11451, Saudi Arabia; 2 Faculty of Health and Life Sciences, Coventry University, James Starley Building, Priory Street, Coventry, CV1 5FB, UK; E-Mail: aa8396@coventry.ac.uk; 3 School of Human Sciences, Newman University College, Birmingham, Genners Lane, Bartley Green, Birmingham B32 3NT, UK; E-Mail: p.collins@newman.ac.uk; 4 College of Education, Qatar University, P.O. Box 2713, Qatar; E-Mail: alnakeeb@qu.edu.qa; 5 Department of Rehabilitation Sciences, College of Applied Medical Sciences, King Saud University, P.O. Box 6941, Riyadh 11452, Saudi Arabia; E-Mail: hsobayel@ksu.edu.sa; 6 School of Health Services, Ministry of Education, Eastern Province 31952, Saudi Arabia; E-Mail: n_abahussain@hotmail.com; 7 Nutrition and Health Studies Unit, University of Bahrain, P.O. Box 32038, Manama, Bahrain; E-Mail: amusaiger@gmail.com; 8 Arab Center for Nutrition, P.O. Box 26923, Manama, Bahrain; 9 Department of Physical Education and Sport Sciences, University of Limerick, Limerick, Ireland; E-Mail: mark.lyons@ul.ie; 10 School of Performing Arts and Leisure, University of Wolverhampton, Gorway Road, Walsall, WS1 3BD, UK; E-Mail: a.m.nevill@wlv.ac.uk

**Keywords:** adolescents, British, culture, dietary habits, lifestyle factors, physical activity, Saudi, screen time, sedentary behaviors

## Abstract

This study investigated the cross-cultural differences and similarity in health behaviors between Saudi and British adolescents. A school-based cross-sectional study was conducted at four cities in Saudi Arabia (Riyadh and Al-Khobar; N = 1,648) and Britain (Birmingham and Coventry; N = 1,158). The participants (14–18 year-olds) were randomly selected using a multistage stratified cluster sampling technique. Measurements included anthropometric, screen time, validated physical activity (PA) questionnaire and dietary habits. The overweight/obesity prevalence among Saudi adolescents (38.3%) was significantly (*p* < 0.001) higher than that found among British adolescents (24.1%). The British adolescents demonstrated higher total PA energy expenditure than Saudi adolescents (means ± SE = 3,804.8 ± 81.5 *vs.* 2,219.9 ± 65.5 METs-min/week). Inactivity prevalence was significantly (*p* < 0.001) higher among Saudi adolescents (64%) compared with that of British adolescents (25.5%). The proportions of adolescents exceeding 2 h of daily screen time were high (88.0% and 90.8% among Saudis and British, respectively). The majority of Saudi and British adolescents did not have daily intakes of breakfast, fruit, vegetables and milk. MANCOVA showed significant (*p* < 0.05) gender by country interactions in several lifestyle factors. There was a significant (*p* < 0.001) gender differences in the ratio of physical activity to sedentary behaviors. In conclusion, Saudi and British adolescents demonstrated some similarities and differences in their PA levels, sedentary behaviors and dietary habits. Unhealthy lifestyle behaviors among adolescents appear to be a cross-cultural phenomenon.

## 1. Introduction

The importance of developing healthy lifestyle habits beginning at childhood and adolescence is quite evident, as diet and physical activity appear to play important roles in maintaining health and preventing diseases [1]. Unhealthy lifestyle behaviors, such as improper diet, physical inactivity, and use of tobacco, are leading causes of preventable chronic diseases worldwide [2,3].

Adolescence is a dynamic yet critical developmental period with short and long-term health implications [4]. Rapid physical growth during adolescence creates an increased demand for nutritional requirement. Recent research also indicates that during adolescence physical activity sharply declines [5] and sedentary behaviors significantly increase [6]. It is now well recognized that physical inactivity and sedentary behaviors are considered independent contributors to the development of chronic diseases and overweight and obesity [7,8]. Consequently, this period provides an important opportunity for the development of health enhancing behaviors among adolescents, especially in light of research findings showing that dietary habits [9] and physical activity [10] have the tendency to track from adolescence to adulthood.

Among adult populations, ethnicity may influence the presence of non-communicable disease risk factors. Higher incidence of diabetes, hypertension, stroke, overall mortality or behavioral risk factor profiles were reported among African Americans [11] and Latinos compared with Whites [12]. Despite having increased risk factors status, ethnic minority groups were found to engage in less frequent leisure-time physical activities than adults in the rest of the population [13]. Among children and adolescents of different ethnicities, differences in physical activity participation have also been documented [14,15]. Furthermore, Arab females, due to cultural and social reasons, have generally fewer opportunities compared with males to engage in leisure-time physical activity, both inside and outside the school, which make it interesting to present similarities and differences from a gender perspective between these two countries.

Furthermore, ethnic differences in food selection and frequency were found to contribute to the marked variations in nutrient intakes between Pacific, Maori, Asian and European adolescents in New Zealand [16]. However, early research showed that ethnicity alone may not be sufficient or adequate in explaining variations in health behaviors of 12-year-old students and that many complex factors including personal, cultural, social and ethnic elements form the behaviors and attitudes of young people [17]. Recent research, indeed supported such earlier findings and concluded that cultural, social and environmental factors during childhood are believed to influence lifestyle habits [18]. Comparison of health-promoting behaviors between Taiwanese and American adolescents also showed that the Taiwanese adolescents had better diet habits and less exercise engagement than the American counterparts [19].

Research comparing dietary habits and lifestyles of Arab adolescents with adolescents in western countries are lacking. Therefore, the objective of the present study was to investigate the cross-cultural differences and similarity in health behaviors, including screen time, physical activity and dietary habits, between Saudi and British adolescents living in urbanized areas of the two countries. The two countries are diverse in terms of cultural, social and environmental characteristics. This study will add to the limited research related to urban youth’s lifestyle behaviors across different ethnic, cultural and environmental backgrounds. 

## 2. Methods

### 2.1. Locations and Participants

The study was carried out in four major urbanized cities in Saudi Arabia and Britain. Saudi Arabian samples came from Riyadh, which is the capital city of Saudi Arabia and located in the central part of the country and from Al-Khobar, which is a modern city located on the eastern coast of Saudi Arabia. Participating cities from Britain were Birmingham and Coventry; both are in central England with Birmingham being the second largest city in the United Kingdom. According to the International Monetary Fund (IMF) 2011 database, the gross domestic products per capita was 21,196 and 38,811 USA dollars for Saudi Arabia and United Kingdom, respectively [20]. 

Data were derived from the Arab Teens Lifestyle Study (ATLS) conducted in Saudi Arabia [21,22] and similar research conducted in Britain [23]. ATLS is a school based, cross-sectional multicenter lifestyle project and detailed description of ATLS can be found elsewhere [21,22]. Data collection took place during the school years 2009/2010 and 2010/2011 in Saudi Arabia and Britain, respectively. The study protocol and procedures were approved by the respective institutional ethical approval committees in both Saudi Arabia and Britain and were conducted in according with international ethical guidelines. In addition, schools permission and students/parents consent for conducting the survey were obtained.

A multistage stratified random-sampling technique was employed to select the participants from the secondary schools in the participating four cities. At first stage schools were randomly selected from the four geographical areas of each participating city. Then, classes were randomly chosen from each grade of the three secondary school grades. The number of selected classes from Saudi Arabia and England were 48 and 36 classes, respectively. Detailed sampling was published elsewhere [22,23]. Unified written instructions were applied to the data collection in both countries. The participants who took part in this survey were free of any major health problems. In the current paper, only adolescents between the ages of 14 and 18 years were included in the analyses, with a total number of 2,806 participants (1,648 from Saudi Arabia and 1,158 from Britain).

### 2.2. Anthropometric Measurements

All measurements were performed in the morning by a trained researcher according to written standardized procedures. Body weight was measured to the nearest 100 g using calibrated portable scales (Seca Ltd., Hamburg, Germany) while the participants was wearing minimal clothing and without shoes. Height was measured to the nearest centimeter using a calibrated measuring rod while the participants was in a full standing position without shoes. Body mass index (BMI) was calculated as the ratio of weight in kg divided by the squared height in meters. The International Obesity Task Force (IOTF) age- and sex-specific BMI cutoff reference standards were used to identify overweight and obese adolescents between the ages of 14 and 17 years [24]. For participants aged 18 years, we used the adult cut-off points of 25–29.9 kg/m^2^ to define overweight and 30 kg/m^2^ and higher for obesity.

### 2.3. Assessment of Health Behaviors

A validated self-report questionnaire was used to assess physical activity levels, sedentary behaviors and dietary habits of the adolescents. The instrument has been previously evaluated for reproducibility and validity and was found to have high reliability and acceptable validity [25]. The questionnaire was translated from Arabic to English by one of the authors (YA) who is bilingual. The translation was reviewed and confirmed by another bilingual author of this paper (HMA). The adaptation of the questionnaire from Arabic to English language and the translation/back translation procedures were conducted according to acceptable methods. When translating the questionnaire to English, every effort was made to have the questionnaire items as conceptually and functionally equivalent. 

A pilot study with a sample of British youth 15–18 years was conducted to assess the cross-cultural suitability of the questionnaire. Focus group discussions with the youth thereafter demonstrated the appropriateness of the questionnaire and confirmed that youth understood each of the questions. No modifications were required, thus construct validity was assumed. All the participants completed the questionnaire in their classrooms under the supervision of their teachers and in front of at least one of the research assistants.

The physical activity part of the questionnaire measures information on frequency, duration and intensity of a variety of light-, moderate- and vigorous-intensity physical activities during a typical week and across different activity domains (transport, household, fitness and sports activities). Moreover, physical activities were assigned metabolic equivalent (MET) values based on the compendium of physical activity [26] and the compendium of physical activity for youth [27]. The participants physical activity levels were then classified into three categories based on cut-off values for total METs-min/week; low active (<1,680 MET-min/week), sufficiently active (1,680–2,519 METs-min/week) and high active (2,520 METs-min/week or more). The cut-off score of 1,680 METs-min/week is an equivalent to 60 min of moderate-intensity daily physical activity (60 min × 7 days × 4 METs = 1,680 METs-min/week), while the cut-off score of 2,520 METs-min/week is an equivalent to 60 min of moderate-to vigorous- intensity daily physical activity (60 min × 7 days × 6 METs = 2,520 METs-min/week). 

Adolescent’s dietary habits were assessed through a number of eating habits frequency questions related to how many times in a typical week the participants consumed breakfast, vegetables (cooked and uncooked), fruit, milk and dairy products, sugar-sweetened drinks including soft beverages, donuts and cakes, candy and chocolate, energy drinks and fast foods. These questions covered healthy as well as unhealthy dietary habits. The students had a choice of answers, ranging from zero intake (never) to a maximum intake of 7 days per week (every day). Participants were then classified into three categories based on the frequency of their intake for each respective food (<3 days/week, 3–4 days/week and >5 days/week). Finally, sedentary behaviors were assessed using typical daily time spent viewing TV, playing video/electronic games, and computer and internet use. For the total screen time cut-off points, we used the American Academy of Pediatrics guidelines of a maximum of 2 h/day [28]. 

### 2.4. Statistical Analysis

Data in each participating city were checked and entered into a computer using standardized entry codes written on an SPSS (SPSS, Inc, Chicago, IL, USA) data file. The current analysis included age range between 14 and 18 years. There was no evidence of data clustering, as intra-class correlation coefficients were very low and were insignificant (*p* value averaged 0.481 in Saudi data and 0.488 in British data). Data were then analyzed using SPSS, version 15. Descriptive statistics were presented as means, standard deviations (or standard errors) and proportions. Data that were not normally distributed, such as physical activity scores in METs-min per week, were log-transformed before performing parametric analysis. Differences in anthropometric measurements, sedentary behaviors, physical activity and dietary habits between countries relative to gender were tested using two-way MANCOVA controlling for the effect of age. The MANCOVA strategy was chosen to be a most appropriate mean by which to examine group differences on multiple dependent variables. It also allowed us to adjust for the potential effects of age on the results and assists in controlling Type I error. For testing the difference in obesity prevalence between Saudi and British adolescents and the differences in the proportions of participants exceeding specific cut-off scores in sedentary behaviors, physical activity and dietary habits, Chi-Square tests were used. The level of significance was set at 0.05 or less.

## 3. Results

Table 1 presents the anthropometric characteristics of the participants stratified by gender and country. Adolescents from Britain were significantly younger and taller than adolescents from Saudi Arabia. On the other hand, Saudi adolescents were significantly heavier and had significantly higher mean BMI values than their peers from Britain. The prevalence of overweight and obesity among Saudi adolescents was significantly (*p* < 0.001) higher than British adolescents. The combined prevalence of overweight/obese among Saudi adolescents (38.3%) was significantly (*p* < 0.001) higher than those found among British adolescents (24.1%). MANCOVA results showed significant gender by country interaction effects for weight (*p* < 0.001), height (*p* = 0.027) and BMI (*p* = 0.025).

**Table 1:** Anthropometric characteristics of Saudi and British adolescents.

Variable	Male		Female		All		*p-*value
	Saudi	British	Saudi	British	Saudi	British	
Number of participants	797	590	851	568	1648	1158	
Age (year) ^a^	16.5 ± 1.0	15.2 ± 0.96	16.4 ± 0.95	15.2 ± 0.95	16.5 ± 0.98	15.2 ± 0.95	country: 0.010 gender × country interaction: 0.599
Weight (kg) ^b^	69.1 ± 20.3	64.4 ± 13.9	58.3 ± 15.1	57.8 ± 11.5	63.5 ± 18.6	61.2 ± 13.3	country: 0.403 gender × country interaction: 0.001
Height (cm) ^b^	168.3 ± 7.6	171.6 ± 8.2	157.2 ± 5.9	161.8 ± 7.6	162.6 ± 8.8	166.9 ± 9.3	country: <0.001 gender × country interaction: 0.027
BMI (kg/m^2^) ^b^	24.3 ± 6.7	21.8 ± 4.0	23.6 ± 5.8	22.1 ± 4.0	23.9 ± 6.3	22.9 ± 4.0	country: <0.001 gender × country interaction: 0.025
Overweight (%) ^c^	19.3	18.2	21.9	17.7	20.7	17.9	<0.001
Obesity (%) ^c^	22.6	6.1	12.9	6.3	17.6	6.2	<0.001
Overweight or obesity (%) ^c^	41.9	24.3	34.8	24.0	38.3	24.1	<0.001

Notes: Data are means and standard deviations or percentages; BMI = body mass index; **^a^** Two-way ANOVA tests (Gender by country); **^b^** Two-way MANCOVA tests of between subjects effects, controlling for the effect of age; **^c^** Chi-Square tests.

The amounts of activity energy expenditure in METs-min/week for the Saudi and British adolescents across different types of physical activities are shown in Table 2. MANCOVA tests revealed significant (*p* < 0.001) gender by country interaction effects in the majority of activities shown in Table 2. In addition, British adolescents demonstrated higher levels of physical activity than Saudi adolescents in almost all the selected physical activities. However, Saudi adolescents expended significantly (*p* < 0.05) more METs-min/week in swimming than the British adolescents did. Total energy expenditure (METs-min/week) resulting from the sum of all types of physical activities was significantly (*p* < 0.05) higher among British compared with Saudi adolescents.

**Table 2:** Physical activity among Saudi and British adolescents.

Variable	Male		Female		All	
	Saudi	British	Saudi	British	Saudi	British
Walking ^a, b, c, d^	351.5 ± 19.1	580.9 ± 22.4	187.9 ± 7.2	527.9 ± 20.6	271.6 ± 11.5	554.6 ± 15.2
Stair Stepping ^a, b, c^	123.4 ± 3.4	183.4 ± 6.0	129.6 ± 3.3	202.7 ± 5.9	126.4 ± 2.4	192.9 ± 4.3
Jogging ^b ,c, d^	444.7 ± 30.6	812.5 ± 44.3	176.1 ± 15.4	373.1 ± 28.4	313.6 ± 17.7	594.7 ± 27.2
Cycling ^a, b, d^	148.6 ± 20.4	284.4 ± 31.9	72.3 ± 9.8	42.7 ± 6.6	111.3 ± 11.5	164.6 ± 16.8
Swimming ^b, c, d^	291.8 ± 23.4	123.7 ± 14.0	193.8 ± 21.9	107.6 ± 15.7	243.9 ± 16.1	115.7 ± 10.5
Martial art ^b, c, d^	114.6 ± 18.8	296.4 ± 29.7	28.3 ± 7.8	73.1 ± 13.4	72.5 ± 10.4	185.7 ± 16.7
Weight training ^a, b, c, d^	204.5 ± 23.2	415.3 ± 30.4	15.3 ± 3.2	65.6 ± 9.8	112.1 ± 12.2	241.9 ± 16.9
Household ^b, c^	107.6 ± 10.4	194.5 ± 14.3	250.8 ± 18.2	397.9 ± 22.4	177.5 ± 10.5	295.3 ± 13.6
Moderate-intensity sport games ^a, b, c, d^	250.7 ± 17.1	500.5 ± 25.0	85.7 ± 10.8	436.5 ± 23.1	170.2 ± 10.5	468.8 ± 17.0
Vigorous-intensity sport games ^b, c^	1,072.8 ± 55.1	1,423.3 ± 60.4	146.9 ± 15.9	549.7 ± 37.9	620.7 ± 31.8	990.3 ± 38.0
Sum of all moderate-intensity physical activity ^b, c, d^	1,001.6 ± 42.0	1,399.6 ± 40.6	718.2 ± 33.6	1,469.9 ± 43.8	863.2 ± 27.3	1,434.5 ± 29.9
Sum of all vigorous-intensity physical activity ^b, c, d^	2,108.6 ± 85.3	3,415.2 ± 109.5	568.6 ± 30.4	1,306.9 ± 61.4	1,356.6 ± 50.5	2,370.4 ± 70.4
Total physical activity ^ a, b, c^	3,110.3 ± 107.9	4,814.8 ± 124.7	1,286.8 ± 52.5	2,776.9 ± 85.1	2,219.9 ± 65.5	3,804.8 ± 81.5

Notes: Data are means and standard errors; Two-way MANCOVA tests controlling for the effect of age: significant differences at *p* < 0.05 for the effect of age (**a**), gender (**b**), country(**c**), and gender by country interaction (**d**).

**Figure 1:**
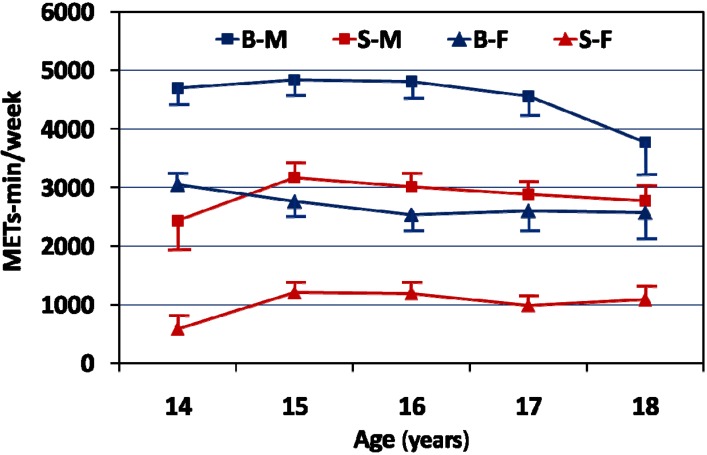
Total energy expenditure from physical activity among Saudi and British adolescents.

Notes: Data are means and standard errors; B-M = British male, B-F = British female, S-M = Saudi male, and S-F = Saudi female.

Figure 1 illustrates the total activity energy expenditure among the Saudi and British adolescents. There is a tendency for physical activity to decline with advancing age among British adolescents though the patterns between males and females were different. However, Saudi adolescents showed similar physical activity patterns relative to age, though lower activity levels among the Saudi females. 

The proportions of Saudi and British adolescents who were classified as low active, sufficiently active and high active are shown in Table 3, relative to age group. Inactivity prevalence was significantly (*p* < 0.001) higher among Saudi adolescents (64%) compared with that of British adolescents (25.4%). Those adolescents who were classified as high active among British adolescents (57.9%) significantly (*p* < 0.001) exceeded that of Saudi adolescents (25.5%). There were slight differences in inactivity prevalence relative to age group in each country.

**Table 3:** Proportions (%) of Saudi and British adolescents classified as low active, sufficiently active and high active.

Activity Category *	Age category	Male		Female		All	
		Saudi	British	Saudi	British	Saudi	British
**Low active** (<1,680 METs-min/week)	14–16	45.2	17.8	76.9	33.7	61.9	25.8
	>16–18	47.9	14.3	83.8	35.2	66.0	23.9
	14–18	46.6	17.3	80.3	34.0	64.0	25.4
**Sufficiently Active** (1,680–2,519 METs-min/week)	14–16	12.2	8.4	11.0	24.2	11.6	16.2
	>16–18	11.9	15.9	7.0	24.1	9.4	19.7
	14–18	12.0	9.3	9.0	24.1	10.5	16.6
**High Active** (≥2,520 METs-min/week)	14–16	42.6	73.8	12.1	42.0	26.5	58.0
	>16–18	40.2	69.8	9.2	40.7	24.6	56.4
	14–18	41.4	73.4	10.7	41.9	25.5	57.9

Notes: * 1,680 METs-min/week = 60 min × 7 day/week × 4 METs (moderate-intensity physical activity), while 2,520 METs-min/week = 60 min × 7 days/week × 6 METs (moderate- to vigorous-intensity physical activity); Chi-Square tests for the differences in physical activity categories between countries: *p* < 0.001.

Table 4 exhibits the mean and standard deviation values of the sedentary behaviors (screen time). Although there was no difference in TV viewing time between the adolescents of the two countries, time spent on computer use indicated a significant (*p* < 0.05) gender by country interaction. In addition, females, especially from Saudi Arabia, spent significantly (*p* < 0.05) more time using a computer than males (3.04 *vs.* 2.78 hours/day).

**Table 4:** Sedentary behaviors among Saudi and British adolescents.

Variable	Male		Female		All	
	Saudi	British	Saudi	British	Saudi	British
TV viewing ^b^	2.70 ± 2.0	2.70 ± 1.7	3.00 ± 2.1	2.87 ± 1.7	2.85 ± 2.0	2.78 ± 1.7
Computer use ^b, c, d^	2.62 ± 2.2	2.68 ± 1.8	3.49 ± 2.5	2.88 ± 1.8	3.04 ± 2.4	2.78 ± 1.8
Total screen time ^b, c, d^	5.31 ± 3.1	5.38 ± 2.8	6.48 ± 3.3	5.75 ± 2.7	5.89 ± 3.3	5.57 ± 2.7

Notes: Data are means and standard deviations; Two-way MANCOVA tests controlling for the effect of age: significant differences at *p* < 0.05 for the effect of age (**a**), gender (**b**), country(**c**), and gender by country interaction (**d**).

The differences in dietary habits between Saudi and British adolescents are shown in Table 5. Results of MANCOVA showed significant (*p* < 0.05) gender by country interactions in the consumption of fruit, milk and dairy products, French fries/potato chips and sweets. Furthermore, the results revealed significantly (*p* < 0.05) higher intake frequency of fruit, milk and dairy products, French fries/potato chips, cake/donuts and energy drinks among British adolescents. 

**Table 5:** Dietary habits of Saudi and British adolescents.

Variable	Male		Female		All	
	Saudi	British	Saudi	British	Saudi	British
Breakfast consumption (frequency /week) ^a, b^	4.17 ± 2.7	4.34 ± 2.7	3.53 ± 2.6	3.35 ± 2.8	3.84 ± 2.7	3.84 ± 2.8
Vegetables consumption (frequency/week)	3.70 ± 2.4	3.87 ± 2.2	3.69 ± 2.4	4.12 ± 2.2	3.69 ± 2.4	4.00 ± 2.2
Fruits consumption (frequency /week) ^a, b, c, d^	3.28 ± 2.3	3.54 ± 2.2	2.45 ± 2.1	3.56 ± 2.2	2.85 ± 2.2	3.55 ± 2.2
Milk/dairy products intake (frequency/week) ^b, c, d^	4.49 ± 2.4	4.56 ± 2.1	3.86 ± 2.5	4.32 ± 2.1	4.16 ± 2.5	4.44 ± 2.1
Sugar-sweetened drinks intake (frequency /week) ^b, c^	4.96 ± 2.2	3.85 ± 2.3	4.44 ± 2.3	3.34 ± 2.3	4.69 ± 2.3	3.60 ± 2.3
Fast foods intake (frequency/week) ^b^	2.90 ± 1.9	2.63 ± 1.8	2.60 ± 1.8	2.57 ± 1.8	2.74 ± 1.9	2.60 ± 1.8
French fries/potato chips intake (frequency /week) ^b, c, d^	2.36 ± 2.0	3.00 ± 1.9	2.90 ± 2.0	2.93 ± 1.9	2.64 ± 2.0	2.96 ± 1.9
Cake/donuts intake (frequency/week) ^b, c^	2.52 ± 2.1	3.10 ± 2.0	2.76 ± 2.1	3.28 ± 2.0	2.65 ± 2.1	3.19 ± 2.0
Sweets intake (frequency/week) ^b, d^	3.04 ± 2.3	3.56 ± 2.1	4.08 ± 2.3	3.86 ± 2.0	3.58 ± 2.3	3.71 ± 2.0
Energy drinks intake (frequency/week) ^b, c^	1.48 ± 2.1	2.23 ± 2.2	0.89 ± 1.7	1.42 ± 2.0	1.17 ± 2.0	1.82 ± 2.2

Notes: Data are means and standard deviations; Two-way MANCOVA tests controlling for the effect of age: significant differences at *p* < 0.05 for the effect of age (**a**), gender (**b**), country (**c**), and gender by country interaction (**d**).

On the other hand, the Saudi adolescents had significantly (*p* < 0.05) higher intake frequency of sugar-sweetened drinks compared with the British. There were also weak but significant relationships between total physical activity levels and total screen time in both Saudi (r = −0.056, *p* = 0.040) and British (r = −0.082, *p* = 0.007) adolescents. 

The proportions of Saudi and British adolescents who exceeded certain cut-off values for sedentary behaviors, physical inactivity and dietary habits are shown in Table 6. There were significantly more British than Saudi adolescents who exceeded 2 hours of daily screen time (90.8 *vs.* 88.0%), met daily intakes of breakfast (33.5 *vs.* 28.4%) and fruit (16.5 *vs.* 13.1%) and exceeded 4 or more weekly intakes of French fries/potato chips (35 *vs.* 27.7%), cake/donuts (40.4 *vs.* 27.9%), sweets (50.1 *vs.* 45.8%) and energy drinks (21.5 *vs.* 12.2%). On the other hand, there were significantly more Saudi than British adolescents who were inactive (64 *vs.* 25.5%) and exceeded 4 or more weekly intakes of sugar-sweetened drinks (64.9 *vs.* 48.9). There were some differences in the prevalence between males and females, however, the prevalence rates changed very little when data were adjusted for age.

**Table 6:** The proportions (%) of Saudi and British adolescents exceeding certain cut-off values for sedentary behaviors, physical inactivity and dietary habits.

Variable	Gender	Saudi	British	*p-*value *
Sedentary (>2 hours/day of screen time)	M	84.2	**89.5**	0.003
	F	91.6	92.2	0.374
	**All**	88.0	**90.8**	0.010
Inactive (<1,680 METs-min/week) ^1^	M	**46.5**	17.3	<0.001
	F	**80.3**	33.9	<0.001
	**All**	**64.0**	25.5	<0.001
Daily breakfast intake	M	34.4	**39.4**	0.035
	F	22.8	**27.4**	0.031
	**All**	28.4	**33.5**	0.002
Daily vegetables intake	M	**23.2**	18.7	0.026
	F	22.7	24.4	0.256
	**All**	23.0	21.5	0.194
Daily fruit intake	M	16.6	15.8	0.366
	F	9.8	**17.3**	<0.001
	**All**	13.1	**16.5**	0.008
Daily milk intake	M	**35.4**	30.5	0.032
	F	27.9	26.9	0.360
	**All**	31.6	28.7	0.058
Sugar-sweetened drinks intake (4+ day/week)	M	**70.2**	53.1	<0.001
	F	**60.0**	44.6	<0.001
	**All**	**64.9**	48.9	<0.001
Fast food intake (4+ day/week)	M	**30.0**	24.1	0.010
	F	24.3	24.2	0.514
	**All**	**27.1**	24.2	0.047
French fries/potato chips intake (4+ day/week)	M	23.4	**34.5**	<0.001
	F	31.7	35.6	0.071
	**All**	27.7	**35.0**	<0.001
Cake/donut/biscuit intake (4+ day/week)	M	26.3	**39.4**	<0.001
	F	29.3	**41.5**	<0.001
	**All**	27.9	**40.4**	<0.001
Sweets/chocolates intake (4+ day/week)	M	36.5	**45.9**	0.015
	F	54.6	54.4	0.503
	**All**	45.8	**50.1**	0.015
Energy drinks intake (4+ day/week)	M	15.6	**27.5**	<0.001
	F	9.0	15.5	<0.001
	**All**	12.2	**21.5**	<0.001

Notes: **^1^** Equals to 60 min per day × 7 days/week × 4 METs (moderate-intensity physical activity); * Chi-Square tests; Bold figures indicate significantly higher proportion.

**Figure 2:**
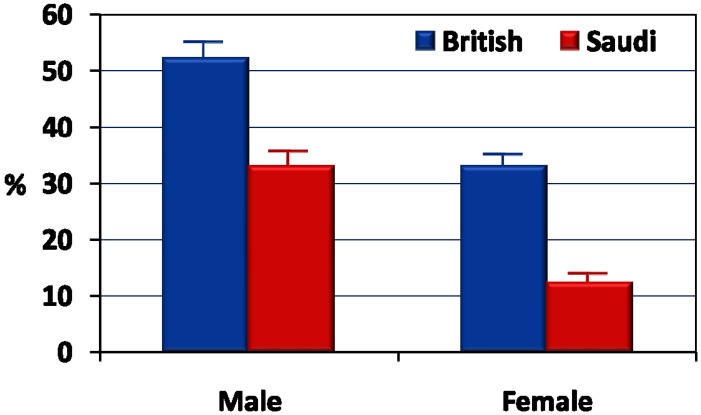
Ratios (%) of physical activity level (hours/day) to sedentary behaviors (screen time in hours/day).

Note: Data are means and standard errors.

Finally, the ratio of physical activity (h/day) to screen time (h/day) was calculated for Saudi and British adolescents stratified by gender. This is shown in Figure 2. ANCOVA tests while controlling for the effects of age, revealed a significantly (*p* < 0.001) higher ratio for the British adolescents compared with the Saudi counterparts. There were also significant (*p* < 0.001) gender differences in the ratio of physical activity to sedentary behaviors. 

## 4. Discussion

In this cross-sectional cross-cultural study, it was found that the prevalence of overweight/obesity and physical inactivity among Saudi adolescents was significantly higher than the British adolescents. There was also a high prevalence of sedentary behaviors among adolescents from both countries. In addition, Saudi and British adolescents demonstrated some similarities and differences in the reported frequency of dietary habits, though the British adolescents exhibited higher percentages of those exceeding certain cut-off values for unhealthy dietary habits. 

The present study corroborated the high prevalence of overweight and obesity among Saudi adolescents that were reported in an earlier study [29]. The overweight and obesity prevalence that was found for British adolescents in the present study is in line with more recent research for British adolescents [30]. Childhood obesity is a growing global epidemic affecting about 10% of the world’s school-aged children [31]. A recent systematic review on obesity trends in children and adults around the world supported an overall leveling off of the obesity epidemic in children and adolescents from Australia, Europe, Japan and the USA [32]. However, the prevalence of obesity among Saudi children and adolescents is still increasing [33].

Findings from the present research show that Saudi adolescents in general and females in particular were less active than their British counterparts. This low activity level is consistent with results previously reported for Saudi children and adolescents [34]. Such high rates of physical inactivity, nevertheless, represent an area of great concern because of the association of inactivity with increased cardiovascular and metabolic risk factors in children and adolescents [35]. It is noteworthy to find that adolescent Saudi females showed the highest risk of physical inactivity; not only as an absolute value but also in relative terms. The female to male physical activity ratio was 41.4% among Saudis and 57.7% among British. Also the Saudi to British physical activity ratio was 64.6% for the males and as low as 46.3% for the females. Culturally, Saudi families may not encourage females to take part in leisure time physical activity.

Saudi Arabia has undergone remarkable economic and social transformations over the past decades. Rapid urbanization, heavy reliance on automobiles, proliferated satellite TV and telecommunication technology and reduced occupational work demands all have contributed to the tremendous lifestyle changes, including increased sedentary behaviors and reduced physical activity [22]. Urbanization and related environmental determinants of physical activity may have acted as a deterrent to active lifestyle in Saudi Arabia. In fact, street networks are engineered to discourage walking among adolescents and young people. This has made people heavily reliant on cars, even for short travel distances. In addition, the hot and dusty environment adds another factor that discourages outdoor physical activity [36]. Conversely, the environment in Britain may be more conducive to outdoor physical activity and whilst the British cities used in this study are heavily urbanized, their urban design shows greater street connectivity, provision of pedestrianized areas and walkways than is the case for Saudi Arabia [23].

According to a study that examined the socio-cultural influences on the physical activity behaviors of culturally and linguistically diverse women living in Australia including Arabic speaking females, physical activity behaviors appeared to be complex and have a strong socio-cultural influence [37]. Differences in physical activity levels due to ethnicity were also reported in 6- to 19-yearr-olds American youth [38]. Moreover, Canadian aboriginals aged 12–17 years were reported to have higher physical activity but lower consumption of vegetables and dairy products than non-aboriginal peers [39]. Several factors that are strongly associated with physical activity in adolescents beside ethnicity were also identified including age, sex, parental support, self-efficacy, previous physical activity, community sports, sensation seeking, parent support, support from others, sibling physical activity, and opportunities to exercise [40].

The present findings showed that boys in both countries were found to be more active than girls. Previous researchers have shown that girls have lower overall activity levels compared with boys [41]. In addition, girls from five European countries spent significantly more time sedentary and less time physically active compared with boys [42]. Females and older youth from the United States were the least active groups [38]. The gender differences in physical activity were found to be greatest during strenuous physical activities [42]. Such findings agree with those of the present study. 

Compared to British adolescents, the present study found a low level of walking among Saudi adolescents. A major contributor to the large difference in walking energy expenditure between Saudi and British adolescents is the low percentage of Saudi school children who walk to and from schools. Results of a study conducted on Saudi boys attending primary schools found that only about 29% of the students walk to and from schools and the corresponding proportion among girls may even be much lower [43]. A previous study, using objective physical activity assessment on European children, has demonstrated that walking to school was significantly associated with higher daily physical activity levels [44].

The current research found that Saudi adolescents expended significantly higher METs-min/week in swimming than the British adolescents (twice the energy expended by the British adolescents). This was an unexpected finding, especially when there is a lack of swimming pools in schools and public parks throughout the countries. However, this can be explained by the fact that most affluent families in Saudi Arabia have indoor swimming pools in their houses. This may explain the higher energy expenditure spent during swimming by the Saudi females compared with their British peers. Also, the hot weather mostly year-round in Saudi Arabia may offer a preference for swimming as leisure-time physical activity. In addition, although the data collection were performed in the Autumn term, the cool weather in England may not favor outdoor swimming as an activity choice. 

The present study showed that 64% of the Saudi and 25.5% of the British adolescents were inactive. Comparison between the present physical activity findings with those from previous studies is somewhat difficult, due to differences in assessment methods, scoring protocol and subject characteristics (age range, sex, ethnicity, and body composition). However, it is important to place the current physical activity findings into perspective. A study conducted in four European countries using objective assessment method found that only 8%–37% of 15-year-old adolescents accumulated at least 60 min of moderate-to-vigorous daily physical activity [45]. Another study involving adolescents from 10 European cities employed an adolescent-adapted version of International Physical Activity Questionnaire (IPAQ) reported much higher mean physical activity (min/day) for males (1,245) and females (1,077) compared with the findings of the present study for Saudi (males: 523.1 and females: 271.8) and British (males: 855.8 and females: 606.6) adolescents [46]. However, the adult version of IPAQ has the tendency to overestimate the level of physical activity [47].

The prevalence of sedentary behaviors (>2 hours/day of screen time) found in the present study among Saudi and British adolescents (88%–90.8%) was remarkably high. Such prevalence is much higher than what was reported for Greek-Cypriot adolescents (52.4%) [48], Finnish boys (48%) and girls (44%) [49], American youth (65% to 71%) [50] and Canadian boys (34%) and girls (41%) [51]. The message stemming from the present study is that there is a need to reduce the time spent by Saudi and British adolescents on TV viewing and computer use. The potential long term adverse health implications of sedentary behaviors on children and adolescents have not yet been determined. However, results of a systematic review on this topic suggested moderate evidence for a longitudinal inverse relationship between screen time and aerobic fitness during childhood [52]. Increased screen time in adolescents was associated with unhealthy dietary habits, including higher intakes of energy-dense snacks, drinks and fast foods as well as lower fruit and vegetable consumption [53]. Furthermore, daily screen time was associated with an increased likelihood of metabolic syndrome among adolescents 12–19 years, independent of physical activity [54].

The weak correlations between physical activity and screen time found in the present study for both samples support the notion that physical activity and sedentary behaviors may really be different entities and were separately linked to distinct dietary habits [7]. Indeed, longitudinal findings showed that changes in TV viewing were not associated with changes in leisure- time moderate-to-vigorous physical activity among 10- to 15-year-old adolescents [55]. Moreover, sedentary activity was found to be significantly and positively associated with insulin resistance while overall physical activity was significantly and inversely associated with insulin resistance [8]. Such findings may imply that to reduce physical activity and sedentary behaviors in youth we need to target somewhat different correlates for each one. 

Saudi females in the present study spent more time on computer games and internet use than males, while there was no gender difference in computer use among British adolescents. This may be partially explained by the fact that females, due to cultural and social reasons, have generally fewer opportunities compared with males to engage in leisure-time physical activity, both inside and outside the school. Thus, they may have more time that can be spent on surfing the internet and connecting with their friends. Similarly, higher use of screen time by females compared with male adolescents was reported for Greek [48] and Canadian youth [51].

The present research found some similarities and differences in the reported frequency of dietary habits among Saudi and British adolescents. Many unhealthy dietary habits were evident among adolescents from both countries. This may reflect the fact that the nutrition and lifestyle transition that have been observed for a while in the developed world are now impacting the developing countries at a much faster rate than expected [56]. During the last few decades, western calorie-dense fast foods and snacks are becoming increasingly accessible and consumed by the young generation in the Middle Eastern countries. Throughout Saudi Arabia and neighboring countries, adolescents have recently been found to have high prevalence of physical inactivity, sedentary behaviors and unhealthy dietary habits along with increased obesity level [22].

Consistent with the literature, the present study findings showed that a significant number of Saudi and British adolescents consumed few fruits and vegetables, with high frequency consumption of sweetened sugar drinks and sweets and chocolates. The Health Behavior in School-aged Children (HBSC) survey, which was conducted during the year 2001–2002 and involved 162,305 pupils of 11, 13 or 15 years of age from thirty-five countries and regions, demonstrated large differences in food habits between countries. The survey showed that the consumption frequency of fruit, vegetables, soft drinks and sweets varied from averages of 2.8 to 5 days/week, 2.4 to 5.5 days/week, 2.1 to 5 days/week and 2.6 to 5 days/week, respectively [57]. Even among European adolescents, variations in food habits exist. A comparison of northern and southern European countries indicated that snacking and consumption of light meals were very common among adolescents in the Scandinavian countries, accounting for 25% to 35% of the daily energy intake [58]. However, snacking and eating out in fast food restaurants appeared to be less frequent among adolescents in the countries of southern Europe compared with those in the United States or in some Nordic countries [59]. In the present study, 35% to 50.1% of the British adolescents consumed French fries/potato chips, cake/donuts/biscuits or sweets and chocolates at a frequency of 4 or more days per week. 

The average consumption (day/week) of fruit (3.55) and vegetables (4.0) by British adolescents found in the present study are almost equivalent to that reported for fruit (3.7) and vegetables (4.1) among adolescents from England in the HBSC survey [57], however, sugar-sweetened beverages and sweets intakes of adolescents in the HBSC study were somewhat higher than those of the British adolescents in the present study (4.4 *vs.* 3.6 days/week and 4.3 *vs.* 3.7 days/week for soda beverage and sweets, respectively). A review of quantitative studies on determinants of fruit and vegetables consumption among children and adolescents revealed that the determinants most consistently supported by evidence are gender, age, socio-economic position, preferences, parental intake and home availability/accessibility [60]. In the present research, no gender differences were observed in the consumption of fruit and vegetables among British children, however, the Saudi males consumed fruits at higher frequency compared to females.

The proportion of Saudi adolescents exceeding four or more intakes of sugar-sweetened drinks was higher than that found among British adolescents, however, more British than Saudi adolescents exceeded four or more intakes of energy drinks. This may be due to the fact that energy drinks have been marketed as consistent with active lifestyle and are placed in vending machines in almost all sports and fitness clubs. Furthermore, they are promoted in sporting events. British adolescents, for just being much more active than Saudi peers, were more likely to have been exposed to the commercial pressure of energy drinks promotion. In addition, the sales of energy drinks in Saudi schools’ canteens are banned. Also, Saudi adolescents are frequently subjected to warning from parents and health educators on the danger of excessive intake of energy drinks. 

The present study is not without limitations. This is a cross-sectional study and does not, therefore, imply any causality. Although every effort was made to minimize over- or under-reporting by the participants, the limitations of assessing dietary habits and physical activity among children and adolescents from different countries using self-reported questionnaire must be considered when interpreting the present study findings. The dietary information obtained from the participating adolescents in this study was based on the frequency of consumption of food items without any regard to quantity or portion size. This may have slightly influenced the information on dietary habits. However, portion sizes are difficult to estimate accurately, especially in people who are not used to the concept of portion size. Another limitation of the study is the lack of pubertal status assessment, so to adjust the prevalence of overweight and obesity to the adolescent’s pubertal stage. Although we feel that the participants were fairly representative of major cities in each of the respective country, it is very difficult to generalize the study results to all teenagers living in large cities, especially in UK. Most major cities in Saudi Arabia with the exception of those in Southwestern Mountains have fairly similar landscape and environmental determinants of physical activity. However, not all British cities have the same provisions for promoting physical activity and their built environments vary greatly. Some cities have little provision in terms of pedestrianized areas, cycle routes and walkways as the urban environment does not suit. UK cities would also vary considerably in terms of walkability, housing density, street intersection density, land use mix, green space and traffic volume. Among the strengths of the current research are the use of highly standardized protocols and procedures in all of the participating centers. In addition, the physical activity questionnaire used in this survey was found to be reproducible, valid and comprehensive, utilizing all domains of physical activity.

## 5. Conclusions

The prevalence of overweight/obesity and physical inactivity among Saudi adolescents was significantly higher than that of the British adolescents. In addition, Saudi and British adolescents demonstrated some similarities and differences in their levels of physical activity, sedentary behaviors and in the reported frequency of dietary habits. Findings from the present study confirm unhealthy lifestyle behaviors, such as increased screen time and unhealthy dietary habits, among adolescents living in urbanized areas. These unhealthy behaviors also appear to be a cross-cultural phenomenon. The high prevalence of sedentary behaviors and physical inactivity among Saudi adolescents, particularly females, are of major public health concern. Future research needs to address the cross cultural determinants of sedentary behaviors, physical activity and inactivity, and unhealthy dietary habits and initiate appropriate interventional programs to combat unhealthy lifestyle habits among adolescents. It is recommended that communities, especially in Saudi Arabia, are designed to promote active transport, ensure safe environments for exercise, and cater to young females within their cultural context. Finally, Saudi females may be a good target for physical activity intervention whereby education about active living and healthy food choices are emphasized.
